# Confronting unequal beginnings in middle- and high-income countries

**DOI:** 10.1038/s41390-025-04017-w

**Published:** 2025-03-20

**Authors:** Lauren J. Klein, Micheal R. DeBaun

**Affiliations:** 1 Pediatric Gastroenterology, Hepatology, and Nutrition, Vanderbilt University Medical Center, Nashville, TN USA; 2 Vanderbilt Institute of Global Health, Nashville, TN USA; 3 Department of Nutrition, University of North Carolina Gillings School of Global Public Health, Chapel Hill, NC USA; 4 Department of Pediatrics, Vanderbilt-Meharry Center of Excellence in Sickle Cell Disease, Vanderbilt University Medical Center, Nashville, TN USA

The first 1000 days of life, from conception to a child’s second birthday, represent a critical window for development and long-term health.^1,2^ During this period, nutrition and exposure to emotional, physical, and environmental stressors can influence rapid growth and brain development. Inadequate nutrition or adverse exposures may impair cognitive development, overall health, and growth during childhood. The Developmental Origins of Health and Disease (DOHaD) framework, originally referred to as the “Barker hypothesis,” suggests that early life disadvantages—including inadequate nutrition and exposure to stressors during fetal development and childhood—also significantly increase the risk of developing chronic diseases, including obesity, diabetes, hypertension, cardiovascular disease, and metabolic disorders.^3^ Brain development happens most rapidly during this window, making it essential to provide adequate nutrition and a supportive environment.^2^ The first 1000 days also present a key opportunity for intervention, as the developing child is highly vulnerable to their conditions, and parents are receptive to guidance.

The initial multisectoral and transdisciplinary strategies to address clinical outcomes in the first 1000 days started in low- and middle-income countries (LMICs).^4^ As this article highlights, low-, middle-, and high-income settings share strikingly similar gaps, opportunities, and root causes of inequity. There is also a pressing need for evidence-informed leadership to drive change. Given these parallels, an important consideration is whether strategies developed in lower-income countries could be adapted to strengthen policies and interventions higher-income countries.^5^ LMICs have often developed cost-effective, community-based solutions out of necessity that could be applied to higher-income settings. Examples relevant to the first 1000 days include health camps and mobile clinics that provide rapid child health screenings and immunizations; simplified nutrition education programs that use culturally relevant food examples to support mothers with limited literacy; mobile health interventions that deliver prenatal care reminders and nutrition advice; community health worker models that offer in-home support for maternal and child health; and affordable infant feeding practices tailored to local food availability. These cost-effective approaches can enhance child health, empower communities, and foster bidirectional learning for global health equity.

The descriptive review article by Del Bono et al. examines health inequalities during this critical period and provides a compelling case for collaborative efforts between governments and healthcare professionals to create interventions in the first 1000 days.^6^ According to the authors, socioeconomic and demographic factors, including a mother’s ethnicity, education, income, employment status, and geographic location, influence maternal and child health. Breastfeeding, the optimal source of infant nutrition, is less common among younger, less-educated, economically disadvantaged, and ethnically marginalized mothers. Similarly, postnatal follow-up for at-risk children, such as preterm infants, is often incomplete, with young maternal age, financial constraints, and inequitable healthcare access disproportionately affecting families from minority backgrounds. Children of lower socioeconomic families have more limited access to vaccinations.

The authors further highlight that child health disparities are closely linked to socioeconomic position (SEP), with lower parental education and income associated with higher mortality rates and developmental delays. The World Health Organization’s 2023 launch of the Health Inequality Data Repository highlights the need to monitor disparities across social determinants of health. Meanwhile, initiatives like the European Child Guarantee aim to break cycles of poverty by ensuring children’s access to essential services. Del Bono et al. advocate that addressing these disparities requires a concerted effort from governments, healthcare systems, and public health professionals to advance equity and improve outcomes during the entire 1000-day period.

Del Bono et al.’s review examines the persistent health inequalities in middle- and high-income countries. To improve clarity and develop evidence-based solutions, it is important to distinguish between health inequalities and inequities, which are often used interchangeably but have different meanings.^7^ Health inequality refers to variations in health or access to healthcare resources resulting from genetics or age. In contrast, health inequity focuses on disparities that are unjust and avoidable. The authors highlight differences in health access and outcomes arising from social, economic, and environmental conditions that systematically disadvantage certain children. The disparities in children’s health outcomes are not merely inequalities; instead, they are inequities—unfair and preventable differences in healthcare outcomes driven by systemic factors.

Despite being a critical window for growth and development, the stages of the first 1000 days, including gestation, the newborn period, and infancy and toddlerhood, are often marked by fragmented care and persistent systemic inequities. Medical care during the 1000 days is often fragmented, as different specialists—obstetricians, neonatologists, and pediatricians—oversee the health of the mother and child at various stages. However, as the authors highlight, the underlying systemic inequities—such as parental employment, transportation, healthcare coverage, housing safety, and access to linguistically appropriate care—remain constant across all stages. The first 1000 days are marked by stress and mental health challenges, including depression and anxiety, which can negatively affect prenatal care, breastfeeding, and parenting practices, further compounding challenges during this critical period.^6^ To address these inequities across the stages of the first 1000 days, integrated care models are needed to promote collaborative care and communication across specialties. The American Academy of Pediatrics suggests a pediatric prenatal visit during the third trimester to establish a medical home, support parents, and identify early risks for the child.^8^ However, broader systemic changes are needed to ensure continuity of care across all stages of the first 1000 days.

Addressing health inequities requires systemic, multisectoral approaches beyond individual-level interventions. Del Bono and colleagues emphasize the need for coordinated efforts among governments, healthcare providers, and communities to reduce health disparities during the first 1000 days. Despite the recognized need for interventions tailored to the most disadvantaged groups, many current strategies remain broad and lack evidence-based approaches. Key proposed strategies include enhancing data collection to identify gaps and evaluate interventions, strengthening maternal and child health services (e.g., home visits, culturally appropriate care), integrating specialty care into combined clinics to improve care coordination and accessibility, and investing in early childhood development through education, nutrition, and parental engagement. However, success demands addressing structural barriers like poverty, discrimination, and social exclusion alongside community-based interventions like peer support and vaccination campaigns. Further efforts should prioritize federal research, surveillance, and targeted programs that equitably improve maternal and child health outcomes.^9,10^

Pediatric academic centers in middle- and high-income countries play a key role in improving outcomes in the first 1000 days. Children’s Hospitals, the epicenter of primary care training, are uniquely positioned to integrate with high-risk pregnancies to provide a novel model of medical care over the first 1000 days. A key component of the transdisciplinary model is establishing best practices for managing high-risk pregnancies and ensuring that the newborn and the mother receive medical care. Arguably, the first step for acknowledging the future mother-child dyad is the now standard practice of transferring high-risk pregnancies to a hospital with a level 3 nursery.^11^ We are optimistic that the leadership of pediatric departments will see the urgent need to be the vanguard for addressing the evidence-based strategies to improve in the first 1000 days of life to improve lifelong health outcomes.

## References

[1] Scott, J. A. The first 1000 days: a critical period of nutritional opportunity and vulnerability. *Nutr. Diet.* **77**, 295–297 (2020). 32478460 10.1111/1747-0080.12617

[2] Darling, J. C., Bamidis, P. D., Burberry, J. & Rudolf, M. C. J. The first thousand days: early, integrated and evidence-based approaches to improving child health: coming to a population near you? *Arch. Dis. Child* **105**, 837–841 (2020). 32111596 10.1136/archdischild-2019-316929

[3] Wadhwa, P., Buss, C., Entringer, S. & Swanson, J. Developmental origins of health and disease: brief history of the approach and current focus on epigenetic mechanisms. *Semin. Reprod. Med.* **27**, 358–368 (2009). 19711246 10.1055/s-0029-1237424 PMC2862635

[4] Keats, E. C. et al. Effective interventions to address maternal and child malnutrition: an update of the evidence. *Lancet Child Adolesc. Health* **5**, 367–384 (2021). 33691083 10.1016/S2352-4642(20)30274-1

[5] Govindarajan, V. & Ramamurti, R. Reverse innovation, emerging markets, and global strategy. *Glob. Strateg. J.* **1**, 191–205 (2011).

[6] Del Bono, C. et al. The first 1000 days: the price of inequalities in high and middle-income countries. *Pediatr. Res.* 1–10 (2025). 10.1038/s41390-025-03880-x 39856230

[7] Bailey, Z. D. et al. Structural racism and health inequities in the USA: evidence and interventions. *Lancet* **389**, 1453–1463 (2017). 28402827 10.1016/S0140-6736(17)30569-X

[8] Yogman, M., Lavin, A., Cohen, G., Committee on Psychosocial Aspects of Child & Family Health. The prenatal visit. *Pediatrics* **142**, e20181218 (2018). 6709439

[9] Hamner, H. C. et al. Improving nutrition in the first 1000 days in the United States: a federal perspective. *Am. J. Public Health* **112**, S817–S825 (2022). 36122314 10.2105/AJPH.2022.307028 PMC9612192

[10] Pérez-Muñoz, C. et al. Interventions in the first 1000 days to prevent childhood obesity: a systematic review and quantitative content analysis. *BMC Public Health* **22**, 2367 (2022). 10.1186/s12889-022-14701-9 PMC9758903 36527103

[11] Hein, H. A. Regionalized perinatal care in North America. *Semin. Neonatol.* **9**, 111–116 (2004). 16256714 10.1016/j.siny.2003.08.009

